# Transfer of a starch phenotype from wild wheat to bread wheat by deletion of a locus controlling B-type starch granule content

**DOI:** 10.1093/jxb/erx349

**Published:** 2017-11-01

**Authors:** Tansy Chia, Nikolai M Adamski, Benedetta Saccomanno, Andy Greenland, Alastair Nash, Cristobal Uauy, Kay Trafford

**Affiliations:** 1 National Institute of Agricultural Biology, Huntingdon Road, Cambridge, UK; 2 John Innes Centre, Norwich Research Park, Norwich, UK; 3 RAGT Seeds Ltd, Grange Road, Ickleton, Essex, UK

**Keywords:** Breeding, B-type starch granules, deletion mutant, grain hardness, granule size distribution, starch granule initiation, starch swelling power, Triticeae, wheat grain

## Abstract

Our previous genetic analysis of a tetraploid wild wheat species, *Aegilops peregrina*, predicted that a single gene per haploid genome, *Bgc-1*, controls B-type starch granule content in the grain. To test whether bread wheat (*Triticum aestivum* L.) has orthologous *Bgc-1* loci, we screened a population of γ-irradiated bread wheat cv. Paragon for deletions of the group 4 chromosomes spanning *Bgc-1*. Suitable deletions, each encompassing ~600–700 genes, were discovered for chromosomes 4A and 4D. These two deletions are predicted to have 240 homoeologous genes in common. In contrast to single deletion mutant plants, double deletion mutants were found to lack B-type starch granules. The B-less grains had normal A-type starch granule morphology, normal overall starch content, and normal grain weight. In addition to variation in starch granule size distribution, the B-less wheat grains differed from controls in grain hardness, starch swelling power, and amylose content. We believe that these B-less wheat plants are the only Triticeae cereals available that combine substantial alterations in starch granule size distribution with minimal impact on starch content.

## Teaser

We describe the production and characterization of bread wheat with grains that lack small B-type starch granules, have near-normal weight and starch content, and have novel grain and starch physicochemical properties.

## Introduction

Whilst much is known about the synthesis of starch polymers in plants, little is known about the determination of starch granule size and shape. Starch granule morphology varies between plant species, and between organs of the same species, and is determined primarily by genetic factors. Members of the Triticeae tribe, which include the economically important cereals wheat (*Triticum aestivum* L.), barley (*Hordeum vulgare* L.), and rye (*Secale cereale* L.) as well as wild wheat or ‘goat grass’ species (*Aegilops* spp), have endosperm starch with a bimodal granule size distribution (Evers, 1973; Parker, 1985; Jane *et al.*, 1994). Individual plastids in the endosperm of these species contain a single, lenticular A-type granule (hereafter, A-granule) and several near-spherical B-type granules (hereafter, B-granules). In Triticeae endosperm cells, a single A-granule initiates early during grain filling in the main body of the plastid. The B-granules form several days later than the A-granules (Parker, 1985; Bechtel and Wilson, 2003) in stroma-filled tubules (stromules) emanating from the plastids (Parker, 1985; Langeveld *et al.*, 2000). Thus, the A- and B-granules found in Triticeae endosperm are the products of two granule initiation events that are separated in time and space. In some growth conditions, a third class of granules, C-type, constituting <4% of the weight of starch, is also observed (Bechtel *et al.*, 1990).

Whilst very little genetic variation is observed between cultivars of domesticated bread wheat (Stoddard, 1999) and barley (Oliveira *et al.*, 1994), a few *Aegilops* species are known to lack B-granules (Stoddard and Sarker, 2000). In a previous publication (Howard *et al.*, 2011), we showed that tetraploid *Aegilops peregrina* and a synthetic tetraploid *Aegilops* with the same genome composition (2*n*=4*x*=28; genomes SSUU) differ in granule size distribution. The synthetic *Aegilops* has both A- and B-type starch granules like most Triticeae, whilst *Ae. peregrina* has A-granules but lacks B-granules. A population segregating for B-granule number was generated by crossing these two lines and was used to study the genetic basis of B-granule content. A combination of bulked segregant analysis and quantitative trait locus (QTL) mapping identified a major QTL located on the short arm of chromosome 4S that accounted for 44.4% of the phenotypic variation. The peak of this QTL was between a conserved orthologous marker called 4G and the end of the short arm of chromosome 4S, and was called *Bgc-1* for B-granule content (Howard *et al.*, 2011).

The segregation of the B-granule trait in our *Aegilops* population showed that lack of B-granules is a genetically recessive trait. This suggests that *Bgc-1* encodes a promotor of B-granules, possibly a promotor of B-granule initiation. It also showed that one active copy per *Aegilops* subgenome is sufficient for B-granules to form and that *Bgc-1* is not required for the formation of A-granules. We presented a model to explain the origins of the B-less polyploid *Ae. peregrina* (Howard *et al.*, 2011), and suggested that B-granule content is likely to be controlled by a single major locus per haploid genome in all Triticeae species.

Here, we have tested the hypothesis that mutation or deletion of all copies of orthologous *Bgc-1* loci in Triticeae genomes, other than *Aegilops*, will result in lack of B-granules in the endosperm. Specifically, we selected deletions of the orthologous *Bgc-1* regions of bread wheat (cv. Paragon) and combined these in a single plant. This resulted in wheat plants with grains containing starch of a unimodal size distribution: the starch has A-granules but lacks B-granules. To our knowledge, these are the first domesticated Triticeae plants that have grains with A-type starch granules only. A preliminary characterization of the growth metrics of the B-granule-less wheat plants, the starch content of the grains, and the physicochemical properties of B-granule-less wheat starch is presented.

## Materials and methods

### Plant material and DNA preparation

Bread wheat (cv. Paragon) deletion mutant lines (M_4_ generation) and DNA (from the M_2_ generation) were obtained from Simon Griffiths and Simon Orford, John Innes Centre, Norwich, UK and were produced and distributed on behalf of the Wheat Genetic Improvement Network (WGIN). The deletions were induced by γ-irradiation as described by Al-Kaff *et al.* (2008). Plants were grown in 1 litre pots in compost (ICL Levington Advance M2 Potting & Bedding compost) in a glasshouse with natural heat and light (during UK summer) or with 20 °C day and 15 °C night and lighting supplemented to give 16 h day length (during UK winter). DNA from these plants was prepared from seedling leaves using a method based on that of Fulton *et al.* (1995).

### Screening for deletion mutants

Two types of PCR markers were designed to screen for DNA deletions (see Supplementary Table S1 at *JXB* online). Homoeologue-specific markers were designed to the bread wheat orthologues of marker 4G, the conserved orthologous gene marker previously shown to flank the QTL for B-granule content in *Aegilops* (Howard *et al.*, 2011). There is a translocation on wheat chromosome 4A from 4A short arm to 4A long arm (Mickelson-Young *et al.*, 1995). Thus, the *Bgc-1* region in the bread wheat A-genome lies on the long arm rather than the short arm of chromosome 4A. Additional homoeologue-specific markers (TC37B, TC30B, and TC34) were designed to wheat genes between 4G and the telomere, and a D-genome-specific marker (4NS5.5) was designed to a gene between 4G and the centromere (Supplementary Table S1). These markers were tested for specificity using the bread wheat cv. Chinese Spring nullisomic/tetrasomic lines N4AT4B, N4BT4D, and N4DT4A (Sears, 1966). Following PCR, the amplification products were separated on agarose gels and stained with ethidium bromide. The absence of a PCR product (or near absence: faint band) was taken to indicate a putative deletion.

An additional homoeologue-non-specific PCR marker, KT71, was designed to a wheat gene in the *Bgc-1* region. This marker was designed to amplify a region of all of the homoeologous genes such that the predicted PCR products were polymorphic in size. There are two KT71 genes on chromosome arm 4AL, therefore four products are amplified by this marker in total (A1, A2, B, and D). The PCR products of KT71 were separated by capillary electrophoresis (Supplementary Fig. S1). The absence of a peak corresponding to one genome (or a very small peak for one genome relative to the others) was taken to indicate a putative deletion.

The PCR markers described above and used for screening were dominant and thus could not distinguish plants that were homozygous wild type from plants that were heterozygous for a deletion. Thus, only homozygous deletion mutants could be uniquely identified.

### Combining the deletion mutants

Seeds (M_4_ generation) of the lines containing putative homozygous deletions (as identified using M_2_ DNA) were grown and screened by PCR as above. Those plants containing confirmed homozygous deletions were grown, allowed to self-fertilize, and deletion mutant seed was collected. The A-genome deletion lines were grown and crossed pairwise with the D-genome deletion lines, in all possible combinations. The F_2_ plants resulting from self-fertilized F_1_ plants were screened using the KT71 marker only, to identify plants with homozygous deletions in both the A- and the D-genomes.

### Analysis of the size and number of deletions in lines A1 and D4

DNA was extracted using a DNeasy Plant Mini Kit (Qiagen), according to the manufacturer’s instructions. DNA libraries and subsequent exome captures were performed at the Earlham Institute, Norwich, UK. For this, the NimbleGen v5.0 protocol (van der Werf *et al.*, 2015) was used with some amendments. A 1000 ng aliquot of DNA was sheared to ~300 bp using a Covaris LE220, and libraries were constructed on a PerkinElmer Sciclone automation platform using a KAPA HTP DNA library preparation kit and a bead-based size selection step. Five cycles of PCR were carried out and either four or five libraries were equimolar pooled to a concentration of 1.4 µg. Each pool of DNA libraries was hybridized at 47 °C for 72 h in a PCR machine with a lid heated to 57 °C. The reaction was optimized and the amount of Universal Blocking Oligos was elevated from 1 µl to 2.8 µl. The *Cot-1* DNA commonly used to block non-specific hybridization was replaced with Developer Reagent and the volume increased from 5 µl to 14 µl. The pull-down and washes were performed on the PerkinElmer Sciclone using an optimized protocol, and the captured DNA received a final 10 cycles of PCR. The resulting libraries were evaluated by capillary electrophoresis (PerkinElmer LabChip GX) to check the quality and by q-PCR/fluorometer (Qubit) to check the quantity. Each pool was run using Illumina v3 chemistry on an Illumina HiSeq 2500 with 100 bp paired-end reads.

The reads were aligned to the IWGSC RefSeq v1.0_parts pseudomolecule reference genome (161010_Chinese_Spring_v1.0_pseudomolecules_parts.fasta; MD5: 10975b9ef5e3ca3784adc128da599cc0) using bwa-mem (version 0.7.12) (Li and Durbin, 2009). Short split hits were marked as secondary alignments for Picard (http://broadinstitute.github.io/picard/) compatibility (option: -M) and a read group header was assigned to each alignment (option: -R). The resulting SAM file was converted to BAM format and sorted using samtools (version 1.3). The MarkDuplicates tool of Picard (version 1.134) was then used to predict PCR and optical duplicates within the sorted BAM file. Suspected duplicate reads were marked (SAM flag 0x400), but not removed from the file. Finally, the output was filtered again to contain only reads mapped in proper pairs (SAM flag 0x2) and then indexed using samtools (version 1.3).

The reads were counted as follows. The multicov tool from the bedtools package (version 2.24.0; Quinlan and Hall, 2010) was used to count the number of alignments falling within 1 Mb bins across each chromosome. Only reads with a mapping quality (MAPQ) of 30 were considered (option: -q 30). Within the output file, the genome co-ordinates of the 1 Mb bins were converted from the RefSeq1.0_parts pseudomolecules to the RefsSeq1.0_full pseudomolecules using a custom bash script. Since the size of the chrXY_part1 scaffolds is uneven, the last bin of each scaffold is smaller than 1 Mb and varies in size between each chromosome. As a result, when combining the two parts of each chromosome, the bins on the second half are shifted by that difference, resulting in uneven start and stop positions. The read counts within each bin were normalized in two steps. First, the read count of each bin was divided by the total number of reads for the given line. Second, this new value was then divided by the corresponding value from the Paragon wild-type line. Thus the read count of each bin is normalized within each line as well as to the Paragon wild type.

Using the normalized data, we determined the positions of large deletions within the mutant lines using the following criteria. A large deletion constitutes a block of at least four adjacent 1 Mb bins, each bin with ≤10% of the read count of the Paragon wild type (i.e. each bin must have a value of ≤0.1). Likewise, the borders of the predicted deletion consist of at least four adjacent 1 Mb bins, each with >10% of the read count of the Paragon wild type; that is, at least four bins must have a value of >0.1 before we consider the deletion finished. The alignments at the borders of the predicted deletions were examined manually to identify the physical position at which coverage in the mutant line drops off.

The normalized data were used to plot whole-genome coverage graphs in R for Windows (version 3.4) using ggplot2 facet_grid (Wickham, 2009). Each chromosome containing a predicted deletion was also plotted separately using ggplot 2. For these plots, we calculated a moving average of the read counts with a window size of 4, in accordance with our criteria to predict a deletion.

### Grain analysis

Grain size, weight, and dimensions were measured using a MARVIN seed analyser (GTA Sensorik, Neubrandenburg, Germany), according to the manufacturer’s instructions. Each grain sample was from a different plant.

### Scanning electron microscopy

Mature grains were fractured in mid-section using a scalpel blade and mounted on the surface of an aluminium pin stub using double-sided adhesive carbon discs (Agar Scientific Ltd, Stansted, Essex, UK). The stubs were then sputter coated with ~15 nm gold particles in a high-resolution sputter-coater (Agar Scientific Ltd) and transferred to a Zeiss Supra 55 VP FEG scanning electron microscope (Zeiss SMT, Germany). The samples were viewed at 3 kV and digital TIFF files were stored.

### Measurement of starch content and extraction of starch

For these experiments, we used grains from three different genotypes: normal (Paragon), control (homozygous *Bgc-1* wild-type F_2_ line, with B-granules), and mutant (homozygous *Bgc-1* mutant F_2_ line, lacking B-granules, sibling of control). Starch content was measured using the Megazyme Total starch assay Kit (AA/AMG) (Megazyme International, Ireland) and the small-scale assay method as described by Trafford *et al.* (2013), except that each extract contained a single grain. For each genotype, four different plants were assayed. For each plant, four grains were extracted and each extract was assayed in duplicate.

For the study of starch physicochemical properties, starch was extracted from additional grains of the same plants as used above for starch content assays. For each of the three genotypes, four replicate starch samples were prepared, each from the grains of a different individual plant. The starch extraction method was modified from South and Morrison (1990). Unless otherwise stated, all procedures were carried out at 4 °C. Ten grains were steeped overnight in 5 ml of water prior to thorough grinding using a pestle and mortar in 20 ml of water. The resulting suspension was filtered through a 100 µm sieve and the filtrate centrifuged for 20 min at 4000 rpm. The pellet was resuspended in 1 ml of water and the suspension layered above 9 ml of 80% (w/v) CsCl in a 15 ml centrifuge tube. This was centrifuged for 15 min at 4000 rpm and the supernatant discarded. The pellet was resuspended in 1 ml of water and transferred to a 1.5 ml microcentrifuge tube prior to centrifugation at room temperature for 5 min at 13000 rpm. The pellet was washed in this way a total of three times with water and then once with ice-cold acetone. Pellets were air-dried and stored at –20 °C.

### Analysis of grain and starch properties

#### Starch granule size analysis

Starch samples were analysed by an image analysis method similar to that used to map *Bgc-1* in *Aegilops* (Howard *et al.*, 2011). This method, rather than laser diffraction particle analysis, was used for two reasons: first because we wished to compare the results for wheat with our previous results for B-less *Aegilops*, and secondly because we had insufficient starch available from the early generations of the F_2_ deletion lines to use laser diffraction.

Two samples of purified starch were placed on a microscope slide and 20 µl of Lugol’s solution (Sigma Aldrich) was added to each, followed by a coverslip. The slide was observed under a light microscope (Leica DM2500) set to bright field with a ×10 objective. From both starch samples on the slide combined, a minimum of 10 representative images were saved as high-resolution jpeg files with scale bars. The images were analysed using ImageJ software (imagej.nih.gov). Having set the scale, images were made binary, the ‘watershed’ function applied (to separate touching granules), and then the ‘analyse particles’ function was applied twice, first with values of 0.785 µm^2^–infinity (to measure the number of particles >1 µm in diameter, i.e. A-+B-granules) and secondly with values of 0.785–78.5 µm^2^ (to measure the number of particles between 1 µm and 10 µm in diameter, i.e. small granules including the B-granules). The results were used to calculate the percentage of small granules per image and the mean values for each starch sample.

#### Near-infrared (NIR) spectroscopy

A FOSS 6500 wavelength scanning near-infrared microscope incorporating ISIScan software was used to measure the protein content, moisture content, and grain hardness for each sample according to the manufacturer’s instructions. Each sample of ~5 g of grain was run in duplicate using a ring cup. The sample spectra were compared with calibration set spectra taken from samples with known protein, moisture, and hardness compositions.

#### Differential scanning calorimetry (DSC)

DSC was performed by Campden BRI (Station Road, Chipping Campden, UK). Three replicate DSC runs were performed per starch sample. The samples were hydrated at a ratio of 2:1 water to starch and left to equilibrate for a minimum of 1 h at ambient temperature. The instrument used was a Mettler Toledo DSC 1. Triplicate aliquots of 30 mg of starch were encapsulated in stainless steel pans and scanned from 25 °C to 115 °C at a rate of 10 °C min^–1^.

#### Swelling power

Approximately 10 mg of starch was added to a pre-weighed, 2 ml round-bottomed tube and the weight of starch recorded to the nearest 0.1 mg. A 1 ml aliquot of water was added to the tube, which was inverted several times before and during incubation in an oven at 80 °C for 30 min. Tubes were allowed to cool to room temperature and then centrifuged at 1500 *g* for 5 min. The supernatant was removed and the tube and pellet were weighed. The weight of the starch pellet was calculated by subtracting the weight of the empty tube. Swelling power was calculated by dividing the weight of the starch plus water after swelling by the starch dry weight.

#### Amylose content

Amylose content was measured in purified starch samples using a method based on that of Knutson and Grove (1994). Approximately 5 mg of starch was added to a 1.5 ml glass vial and the weight of starch recorded to the nearest 0.1 mg. After addition of 50 µl of 3 M CaCl_2_, the vial was capped, vortexed, and incubated at room temperature for 10 min. After addition of 0.5 ml of 6 mM I_2_ in DMSO, the sample was vortexed and incubated whilst rotating slowly at 70–75 °C for 30 min. After vortexing, a 10 µl aliquot was transferred to a fresh glass vial and 100 µl of 6 mM I_2_ in DMSO and 800 µl of water were added. The sample was mixed and incubated at room temperature for 10 min before the absorbance at 600 nm was measured using a glass cuvette and a spectrophotometer. Mixtures of potato amylose and maize amylopectin (Sigma-Aldrich, Poole, UK) containing 0–50% amylose were prepared and assayed as above. Amylose content was estimated with reference to the amylose/amylopectin standard curve.

## Results

### Screening for deletion mutants

Our previous QTL analysis (Howard *et al.*, 2011) showed that a single locus, *Bgc-1*, on the short arm of chromosome 4S in *Ae. peregrina* accounted for 44.4% of the control of B-granule content. The *Bgc-1* locus lies between marker 4G and the telomeric end of the short arm of chromosome 4S. To test whether there is a similar locus responsible for B-granule content in *T. aestivum*, we screened a bread wheat cv. Paragon deletion mutant population for lines carrying deletions in the *Bgc-1*-orthologous regions of chromosome arms 4AL, 4BS, and 4DS.

Out of a population of ~1000 Paragon deletion mutant lines, three lines carried deletions in the A-genome *Bgc-1* region (A1–A3) and five lines carried deletions in the D-genome *Bgc-1* region (D1–D5) (Table 1). No line with a deletion affecting the B-genome *Bgc-1* region was discovered. Of the eight deletion lines, four (A2, A3, D3, and D4) lacked all of the genes tested (Table 1), suggesting that in these lines there is a relatively large-scale deletion affecting the *Bgc-1* region. Three lines (A1, D1, and D2) gave PCR products with marker 4G but not with any of the other markers tested. This suggests that in these lines, the deletion is close to, but does not include, 4G. One line (D5) lacked a PCR product with 4G only, suggesting that the deletion in this line may not include *Bgc-1*. Line D5 was not used in subsequent experiments.

**Table 1.** Genotype of the bread wheat cv. Paragon deletion mutant lines

Chromosome arm	Line	Plant	Marker					
			TC37b	KT71	TC30b	TC34	4G	4N5.5
4AL	A1	1	–	–	–	–	+	NA
4AL	A1	2	Faint	–	Faint	–	+	NA
4AL	A2	1	–	–	–	–	–	NA
4AL	A2	2	Faint	–	–	–	–	NA
4AL	A2	3	–	–	Faint	–	–	NA
4AL	A2	4	–	–	–	–	–	NA
4AL	A3	1	–	–	–	–	–	NA
4AL	A3	2	–	–	–	–	–	NA
4DS	D1	1	–	–	Faint	–	+	ND
4DS	D1	2	–	–	Faint	–	+	ND
4DS	D2	1	–	–	–	–	+	ND
4DS	D3	1	–	–	–	–	–	–
4DS	D4	1	–	–	–	–	–	–
4DS	D4	2	–	–	–	–	–	–
4DS	D4	3	–	–	–	–	–	–
4DS	D4	4	FAINT	–	–	–	–	–
4DS	D5	1	+	+	+	+	–	+
4DS	D5	2	+	+	+	+	–	+
4DS	D5	3	+	+	+	+	–	+

The markers are ordered (left to right) in the order in which they occur on the chromosome arm.

+, PCR positive (gene present); –, PCR negative (gene deleted).

There is no A-genome homoeologue of 4N5.5. This is indicated by NA. Markers not determined are indicated by ND. Faint, low amplification giving faint bands on agarose gels.

### Combining the deletion mutants

To generate plants with deletions in the *Bgc-1* regions of both the A- and D-genomes, mutant lines A1–A3 were crossed pairwise to lines D1–D4, in all combinations. All crosses were successful except for A2×D3. The resulting F_1_ plants were grown and allowed to self-fertilize to generate F_2_ progeny. For each F_2_ family, 24 grains were sown. DNA was prepared from seedling leaves and was PCR screened using the KT71 marker only. Out of a total of 457 F_2_ plants that were successfully screened, only one plant carried homozygous deletions on both 4AL and 4DS (Supplementary Fig. S1). This AD double deletion mutant plant was derived from a cross between lines A1 and D4. The expected proportion of homozygous double mutants in the F_2_ for deletions transmitted without bias is 6% (1/16). The observed proportion of homozygous double mutants in the F_2_ was 0.2%. F_2_ plants with single homozygous deletions on either the A- or D-genomes were also under-represented in the population.

To screen for additional AD double deletion mutant plants, selected F_2_ plants with homozygous single deletions on either 4AL or 4DS were allowed to self-fertilize and the F_3_ seeds were each cut in half. Plants recovered from the embryo halves of the F_3_ seeds were screened for deletions using the KT71 marker. Four additional homozygous double mutant plants were discovered, and the non-embryo half-seeds were phenotyped for starch granule morphology (see below). Unfortunately, only one of these additional double mutants survived and was fertile. This second double mutant plant, like the first double mutant, derived from a cross between mutant lines A1 and D4. The original F_2_ AD double mutant plant and its progeny only were used in subsequent experiments.

To screen for a wild-type segregant control line for the AD double deletion mutant plant, the F_2_ plants that were PCR positive (i.e. wild type or heterozygous) for both deletions were grown and allowed to self-fertilize. An F_2_ plant which gave progeny that were all PCR positive (i.e. homozygous wild type) for both *Bgc-1* deletions was selected.

### The size and location of deletions in Paragon lines A1 and D4

Next-generation sequencing was used to investigate the size and location of deletions in the genomes of Paragon lines A1 and D4. For this, libraries were created and hybridized to a set of exome capture probes before sequencing using an Illumina platform. We aligned the reads to the latest hexaploid wheat genome assembly (IWGSC RefSeq1.0) and counted the number of reads aligning within 1 Mb sized contiguous segments (bins) along the chromosomes. The read counts were normalized both within each mutant line and to the Paragon wild-type line.

**Fig. 1.**
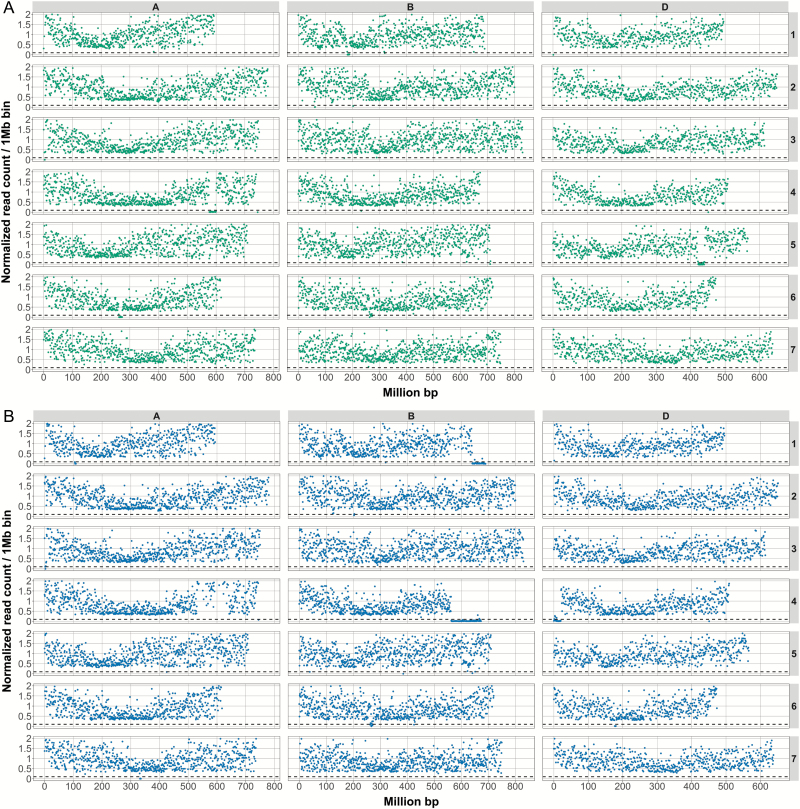
The size and position of deletions in two Paragon mutant lines. The two Paragon deletion mutant lines, A1 (A) and D4 (B), which were crossed to generate the B-granule-less mutant were exome sequenced. The graphs show the number of counts (normalized against Paragon wild type) on the *y*-axis against the chromosomal position (IWGSC RefSeq v1.0) of the read (in million base pairs) on the *x*-axis. The dotted line represents the 0.1 normalized coverage cut-off. For each panel, the rows show chromosomes 1–7 (top to bottom, respectively) and the A-genome data are shown on the left, the B-genome in the middle, and the D-genome on the right. Deletions are indicated by regions of reduced counts. Deletions in line A1 are on Chr 1B, 4A, 5D, and 6A and in line D4 on Chr 1A, 1B, 4B, 4D, 6D, and 7A. (This figure is available in colour at *JXB* online.)

To predict the location of large deletions within the mutant lines, we applied several criteria to the normalized data. First, if the normalized read count within a bin was ≤10% of the level of the wild type, we highlighted said bin. Second, if at least four adjacent bins were highlighted, we considered them to belong to a single large deletion. Third, we considered the deletion to be finished if at least four adjacent bins had a normalized read count of >10% of the level of the wild type. Using these criteria, we predicted four large deletions within Paragon mutant A1 and six large deletions within Paragon mutant D4 (Fig. 1; Table 2). The drop in coverage on chromosome 6B at ~260 Mb in both mutants is due to a natural polymorphism between the background variety (Paragon) and the reference (Chinese Spring) that results in misalignment of reads. We do not consider this region as a bona fide irradiation-induced deletion.

**Fig. 2.**
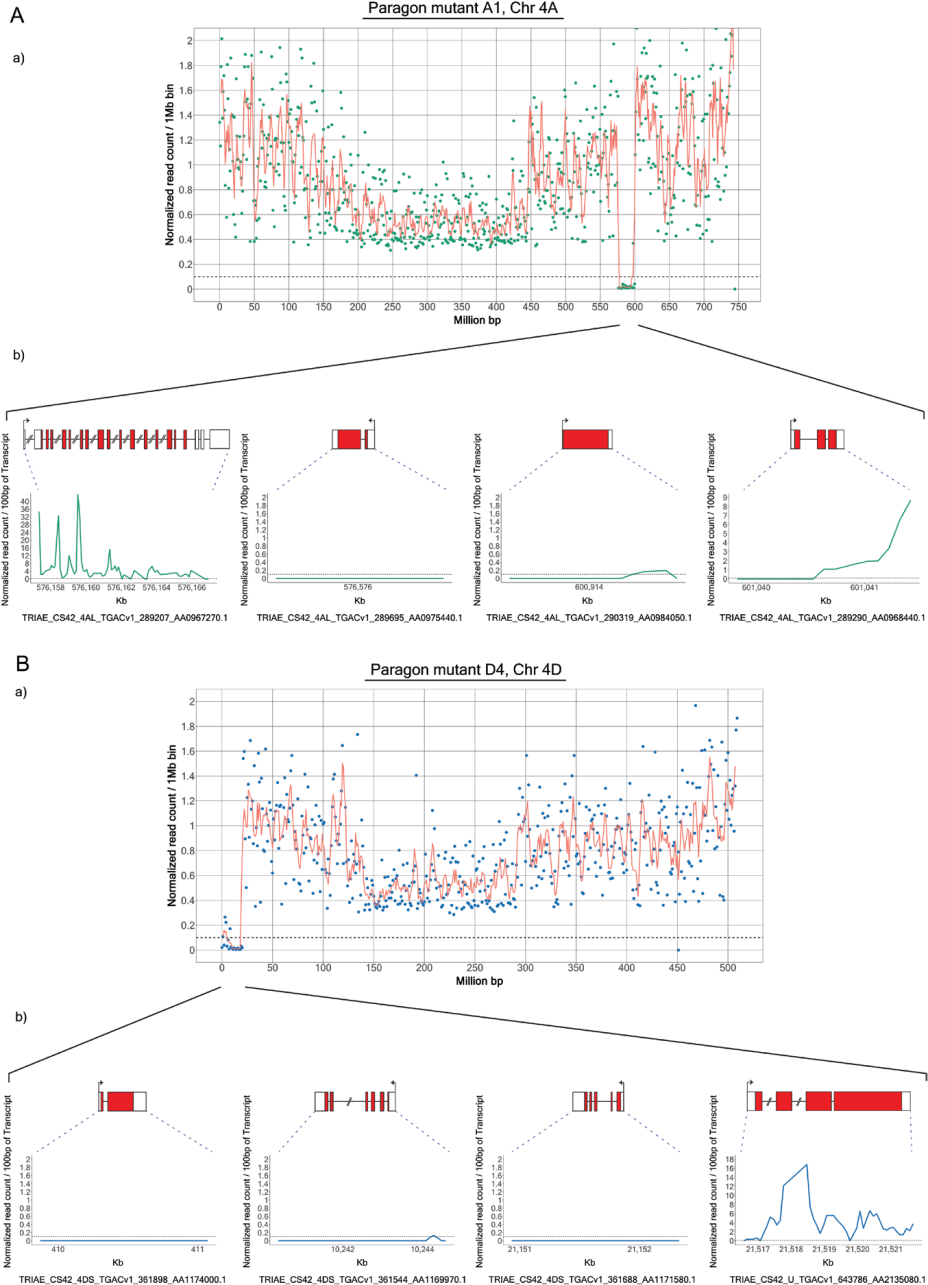
Identification of genes flanking the Chr 4AL and 4DS deletions. The read coverage at the borders of the 4AL and 4DS deletions in lines A1 (A) and D4 (B), respectively, was examined to determine the precise position at which the coverage in the mutant line drops off. The genes closest to the borders of each deletion were identified manually using the TGAC_v1 gene models. In each panel, the graph at the top shows the exome sequencing data for the whole chromosome. The bottom panel in (A) shows the normalized counts for the two genes surrounding the left border and for the two genes surrounding the right border (on the left and right hand sides, respectively). In (B), three gene models distributed along the deletion are shown as well as the closest gene model outside of the deletion. (This figure is available in colour at *JXB* online.)

To better define the extent of the predicted deletions, we examined the alignments at the borders of the deletions to determine the precise position at which the coverage in the mutant line drops off. We then identified the genes closest to the borders of each deletion. For this analysis, we used the TGAC_v1 gene models and only considered transcripts showing coverage in Paragon wild type (Fig. 2; Supplementary Figs S2–S11). Using this approach, the sizes of the deletions increased by up to ~25% compared with the previous method (Table 2), which is partly due to the higher resolution. However, this approach is likely to inflate the size of the deletions artificially, because of our prerequisite regarding coverage, as well as the fact that the set of gene models is incomplete. This is especially evident with the group 6 and group 7 deletions, which increased the most in size. The annotated gene content at the borders of these deletions was low, resulting in this apparent increase in size.

**Table 2.** The approximate size and position of the large deletions

Paragon mutant	Chromosome	Deletion start position	Deletion end position	Approx. size of deletion (bp)
**A:**				
**Line A1**	Chr 1B	182000000	190000000	8000000
	Chr 4A	576555092	600555092	24000000
	Chr 5D	422000000	440000000	18000000
	Chr 6A	262000000	274000000	12000000
**Line D4**	Chr 1A	105000000	110000000	5000000
	Chr 1B	640720154	689851870	49131716
	Chr 4B	563014251	673617499	110603248
	Chr 4D	0	21000000	21000000
	Chr 6D	177000000	181000000	4000000
	Chr 7A	332000000	336000000	4000000
**B:**				
**Line A1**	Chr 1B	181218604	190284536	9065932
	Chr 4A	576167498	601039825	24872327
	Chr 5D	421138884	441103787	19964903
	Chr 6A	260435891	276294537	15858646
**Line D4**	Chr 1A	104949904	110279278	5329374
	Chr 1B	640164065	689851870	49687805
	Chr 4B	562939364	673617499	110678135
	Chr 4D	0	21516171	21516171
	Chr 6D	176481122	181405837	4924715
	Chr 7A	330452708	336755079	6302371

Regions of low coverage (predicted deletions) in Paragon mutant lines A1 and D4 were identified from exome sequencing data. The approximate size and position of the deletions was based on: A, division of the chromosome into 1 Mb sized bins; B, the positions of TGAC_v1 transcripts surrounding the borders of the deletions.

The positions of the markers used to discover the deletions on chromosome arms 4AL and 4DS (Table 1; Supplmentary Table S1) were compared with the positions of the deletions (Fig. 2; Table 2) revealed by exome sequencing (Supplementary Table S2). For both lines, the genes corresponding to the PCR markers all lie within the group 4 deletions predicted by exome sequencing. Thus, the exome sequencing predicts that none of the PCR markers is expected to give a product for either line A1 or line D4. However, a PCR product was observed with marker 4G for line A1. A possible explanation for this discrepancy is that the physical position of 4G in cv. Paragon is different to the reference sequence, IWGSC RefSeq v1.0 for cv. Chinese Spring.

The analyses described above showed that line A1 had four large (≥4 Mb) deletions (on chromosomes 1B, 4A, 5D, and 6A) whilst line D4 had six large deletions (on 1A, 1B, 4B, 4D, 6D, and 7A). Note that the deletion on chromosome 4BL in line D4 is not in the region syntenic to the 4DS/4AL deletions. Thus, exome capture suggests that there are multiple large-scale, γ-induced deletions in each of the lines. The estimated sizes of these deletions ranged from 4 Mb to 110 Mb, the latter constituting a deletion of approximately a sixth of chromosome 4B (Table 2). None of the predicted deletions overlapped between the two lines, suggesting that these are indeed independent mutants. Crucially, each mutant line was predicted to carry at least one deletion on a group 4 chromosome. Based on the IWGSC RefSeq v1.0 physical sequence, there are 679 and 652 predicted TGAC_v1 high and low confidence gene models (Clavijo *et al.*, 2017) in the deletions on chromosome arms 4AL and 4DS, respectively. Of these, 240 genes have homoeologues in both deletions.

### Phenotypes of single and double mutants

**Fig. 3.**
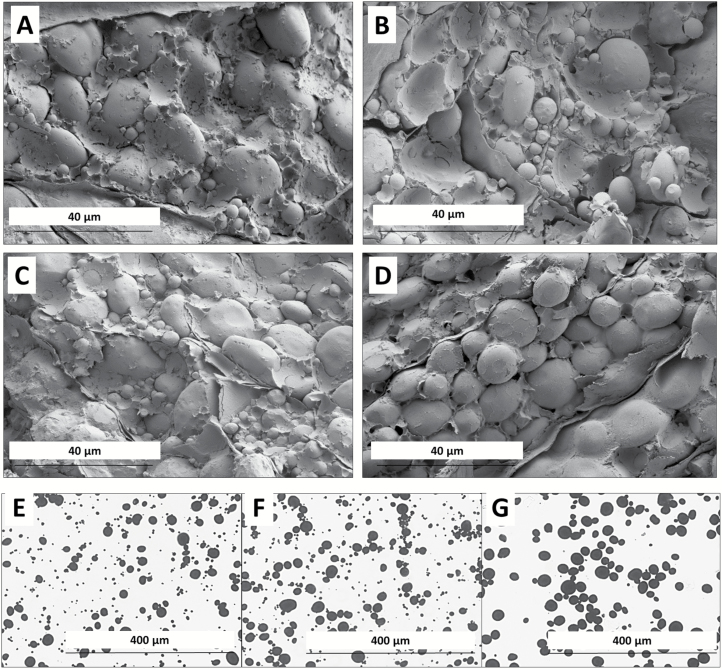
Microscopy of mature endosperm and purified starch granules. (A–D) Mature grains observed by SEM. (A) Wild type: Paragon. (B) A1 single deletion mutant. (C) D4 single deletion mutant. (D) AD double deletion mutant. (E–G) Purified starch observed by light microscopy. (E) Paragon. (F) Control (wild-type segregant). (G) AD double deletion mutant.

The starch granules from the single deletion mutants were examined microscopically and, like the normal wheat cv. Paragon, all were found to possess A- and B-granules (Fig. 3 and data not shown). Starch from the control (wild-type segregant) also had both A- and B-granules (Fig. 3). Starch from the F_2_ AD double mutant line, however, lacked small B-type starch granules when examined by light microscopy or SEM (Fig. 3). The starch granule size distribution in this line was clearly unimodal rather than the normal bimodal distribution. The starch granules in the non-embryo halves of the four additional double mutant plants discovered in the F_3_ screen were also found to lack B-granules. This shows that combining deletions of the *Bgc-1* regions of both 4AL and 4DS in the same plant prevents the formation of, or severely reduces the number of, B-type starch granules in wheat.

The F_3_ progeny of the F_2_ AD double mutant (B-less) plant were grown together with replicate wild-type segregant (control) and wild-type Paragon plants. The height of the primary tillers of both the control and B-less plants was significantly less than that of Paragon (Table 3). However, there was no difference in tiller height between the control and B-less plants.

**Table 3.** Growth metrics and starch characteristics

	‘Normal’ (Paragon)			Control (wild type)			B-less mutant			Student’s *t*-test (*P*-value)		
	Mean	*n*	SE	Mean	*n*	SE	Mean	*n*	SE	Normal versus control	Normal versus mutant	Mutant versus control
**Growth metrics**												
Height of primary tiller (cm)	80.86	18	1.55	72.50	8	3.75	65.08	12	2.37	0.02*	0.00*	0.09
Grain weight (mg)	38.66	18	1.00	31.86	8	2.20	33.94	23	1.02	0.08	0.00*	0.34
Grain length (mm)	6.24	18	0.04	5.64	7	0.15	5.73	26	0.05	0.00*	0.00*	0.51
Grain width (mm)	3.34	18	0.04	3.04	7	0.10	3.19	26	0.04	0.00*	0.01*	0.11
Grain area (mm^2^)	17.03	18	0.29	14.01	7	0.73	14.75	26	0.24	0.00*	0.00*	0.22
**Starch content (% grain weight**)	64.8	4	1.1	57.8	4	2.0	52.0	4	1.8	0.02*	0.00*	0.08
**Starch characteristics**												
***NIR***												
Protein content (% dry matter)	16.44	6	0.48	18.59	3	0.64	17.66	5	1.19	0.03*	0.34	0.59
Moisture content (% by weight)	12.25	6	0.06	11.82	3	0.17	12.15	5	0.11	0.02*	0.44	0.14
Hardness (hardness index)	68.45	6	2.09	63.33	3	1.76	39.62	5	4.21	0.16	0.00*	0.01*
***Granule size***												
Small granule content (% granules 1–10 µm diameter)	53	4	4	55	4	4	17	4	5	0.78	0.00*	0.00*
Area of small granules (µm^2^)	25.54	4	0.66	23.50	4	0.64	38.64	4	5.26	0.07	0.05	0.03*
Area of large granules (µm^2^)	317.8	4	6.6	294.5	4	15.1	349.3	4	23.6	0.21	0.25	0.10
***Amylose content (% starch***)	23	4	1	25	4	1	21	4	1	0.26	0.07	0.01*
***Swelling power***	9.12	4	0.27	9.09	4	0.32	11.37	4	0.39	0.94	0.00*	0.00*
***DSC***												
Enthalpy of starch gelatinization (J g^–1^ solids)	8.13	3	0.11	8.20	3	0.03	7.96	3	0.47	0.58	0.74	0.63
Onset temperature (°C)	57.49	3	0.42	57.25	3	0.28	56.67	3	0.09	0.66	0.13	0.12
Peak temperature (°C)	61.79	3	0.48	61.82	3	0.24	62.34	3	0.40	0.96	0.43	0.33
End temperature (°C)	69.34	3	0.76	70.34	3	0.38	71.74	3	0.21	0.30	0.04*	0.03*
Enthalpy of melting of the amylose–lipid complex	0.39	3	0.02	0.46	3	0.04	0.67	3	0.06	0.24	0.01*	0.04*
Onset temperature (°C)	98.53	3	0.19	98.72	3	0.29	97.52	3	0.18	0.60	0.02*	0.02*
Peak temperature (°C)	103.5	3	0.27	103.9	3	0.22	103.3	3	0.19	0.35	0.47	0.09
End temperature (°C)	108.0	3	0.31	108.8	3	0.25	108.3	3	0.25	0.13	0.55	0.22

All values are expressed as mean ±SE per plant.

*n*, the number of plants measured; swelling power, weight of swollen starch/weight of dry starch. Statistically significant values are indicated by an asterisk.

Grain weight, length, width, and area were compared between the three genotypes (Table 3). As with primary tiller height, the Paragon grains were larger than either the control or B-less mutant grains; however, the difference in grain weight between Paragon and the control was not significant. There was no difference in grain size or weight between control and B-less mutant grains. As for grain weight and size, the starch content of both control and B-less plants was significantly less than that of Paragon, but there was no significant difference in starch content between the control and B-less plants.

This analysis suggests that the lack of B-type starch granules due to deletions in the *Bgc-1* regions specifically, has no detectable effect on plant height, grain size, or starch content. Additional deletions, however, in both control and B-less plants do affect plant growth compared with the normal Paragon line.

### Starch and grain properties

Starch was purified from the grains of the three genotypes: Paragon, control, and the B-less mutant. Several starch and grain properties were examined (Table 3) and, for the following, there were no detectable differences between the control and B-less genotypes: protein content, moisture content, the size of the A-type starch granules, and most of the DSC parameters (enthalpy of starch gelatinization, onset, and peak temperatures). However, some properties were different between control and B-less genotypes. These included grain hardness (B-less were softer), small granule content (defined as starch granules between 1 µm and 10 µm in diameter; B-less had fewer granules <10 µm in diameter), size of the small granules (B-less had larger small starch granules, on average), amylose content (B-less had slightly lower amylose content), swelling power (B-less starch swells more), and, finally, the DSC end temperature for the starch gelatinization peak (B-less end temperature was ~1.5 °C higher).

## Discussion

We hypothesized that wheat and other Triticeae may have a locus controlling B-type starch content on the group 4 chromosomes orthologous to the *Bgc-1* locus identified in *Aegilops* (Howard *et al.*, 2011). To test this, we selected and combined deletions of the orthologous *Bgc-1* regions of bread wheat. By screening a population of deletion mutants created by γ-irradiation of the bread wheat cv. Paragon, we found several deletions in the *Bgc-1* regions of both the A- and D-genomes. No deletions were found in the B-genome. The single genome deletion mutant plants had normal starch granule size distribution. This was expected since in *Aegilops*, we found *Bgc-1* alleles conferring the presence of B-granules to be dominant. However, combining the A- and D-genome deletions together in one plant resulted in grains lacking or having severely reduced numbers of B-type starch granules as judged by light microscopy and SEM. The normal bimodal granule size distribution—with distinctly different sizes and shapes of A- and B-type granules—was replaced in the AD double mutant by a unimodal distribution of granule size. Thus, unexpectedly, deletion of *Bgc-1* on two of the three genomes of wheat is sufficient to eliminate B-granules effectively. This suggests that (i) bread wheat, like *Aegilops*, has *Bgc-1* loci on the group 4 chromosomes that control B-granule content; (ii) there are active *Bgc-1* loci on the A- and D-genomes; and (iii) there may be no active *Bgc-1* locus on the B-genome of wheat. The deletions of the *Bgc-1* regions on 4AL and 4DS were each predicted to contain 600–700 genes. Amongst these are 240 genes with homoeologues in both deletions. We hypothesize that one of these 240 genes is *Bgc-1*, assuming that the gene content in cv. Paragon is similar to that in cv. Chinese Spring.

We found only a few double deletion mutant plants amongst the progeny of crosses between lines A1 and D4. Given the size of the *Bgc-1* deletions present in these lines, it is perhaps not surprising that their transmission from one generation to the next was selected against. Two double deletion mutant lines survived. These were derived from crosses between the same A- and D-genome deletions (A1×D4) but from different F_1_ plants. The mutant lines are therefore independent and this suggests that it is the combination of the two deletions on chromosomes 4A and 4D that is responsible for the lack of B-granules, rather than the combination of any of the other deletions present in lines A1 and D4.

The lack of B-granules was obvious when starch from mature double deletion mutant grains was examined microscopically and was confirmed by quantitative analysis of starch granule size distribution using image analysis (Table 3). Image analysis showed that there are some small granules (with diameters between 1 µm and 10 µm) in the B-less mutant. This is also the case in the B-less *Aegilops* which we had examined previously using a similar method (Howard *et al.*, 2011). The lack of B-granules in B-less species of *Aegilops* was also established by laser diffraction particle size analysis (Stoddard and Sarker, 2000). We hypothesize that the small granules that we observed in the B-less mutant of wheat are small A-granules rather than true B-granules, and this hypothesis is supported by the fact that the average size of this category of granules is larger in the B-less starch than in the wild-type control (Table 3).

The reduction in small granule content in the mutant was not matched by equivalent reductions in starch content or grain weight. In wheat, the contribution of B-type starch granules to the total starch content is estimated to be ~30% (Lindeboom *et al.*, 2004). In contrast, both starch content and grain weight were only marginally decreased in the mutant relative to the control, and neither of these changes was statistically significant (Student’s *t*-test: *P*-value >0.05). This discrepancy implies that in the double-deletion mutant that appears to be unable to initiate B-type starch granules, more starch than normal is being deposited in the form of large A-granules.

Some of the physicochemical properties of starch from B-less wheat grains differ from those of the control with B-granules. This is, in part, predicted from published data on the properties of purified A- and B-granules from normal wheat and barley, some of which vary (Lindeboom *et al.*, 2004). However, the amylose content of the B-less wheat starch was slightly lower than that of the control, whilst the amylose content of purified A-granules has been found to be either greater than (Peng *et al.*, 1999; Takeda *et al.*, 1999; Li *et al.*, 2013) or the same as (Evers *et al.*, 1974; Myllarinen *et al.*, 1998) that of purified B-granules. These data suggest that the A-granules in the B-less mutant bread wheat differ in composition from the A-granules in normal bread wheat. These unexpected physicochemical properties could be related to the increase in starch deposition in the form of A-type starch granules in the B-less mutant relative to that in normal wheat.

In addition, we found that the swelling power of B-less starch was higher than that of the control. Published measurements of the swelling power of A- and B-granules purified from wheat disagree with one another. Li *et al.* (2013) found that the swelling power of A-granules was higher than that of B-type, whilst Wei *et al.* (2010) found the opposite.

In other respects, the physicochemical properties of B-less and control starches did not vary. The gelatinization enthalpy of B-less mutant starch was the same as that of control starch. Our values are within the range expected for wheat starch (7–10 J g^–1^ solids). This result is predicted by the work of Eliasson and Larsson (1983), Ao and Jane (2007), and Wei *et al.* (2010) who showed that the gelatinization enthalpy of wheat starch is independent of the granule size distribution. However, others have found higher gelatinization enthalpies for A-type than for B-type starch granules in wheat (Chiotelli and Le Meste, 2002; Li *et al.*, 2013; Kumar *et al.*, 2016).

We found that the *Bgc-1* deletions were transmitted from one generation to the next with a lower frequency than wild-type chromosomes. This suggests that the deletions may be deleterious and that chromosomes carrying these deletions are selected against. In addition, the deletion mutant plants (both the control and B-less mutant) grew less well than wild-type Paragon. This could be due to the multiple large deletions (other than the deletions of the *Bgc-1* regions) that are scattered throughout the genomes of the parent plants, A1 and D4 (Fig. 2; Table 2), some of which are likely to have been inherited by the control and B-less mutant lines. In the B-less plants, there are many genes in the deleted *Bgc-1* regions in addition to *Bgc-1* itself, and their loss could also contribute to poor performance. The gene at the *Bgc-1* locus responsible for B-granule content has not yet been identified but, when it is, it could be specifically manipulated using sequenced mutants (Krasileva *et al.*, 2017) or genome editing (Zhang *et al.*, 2016) to eliminate B-granules with presumably far fewer (if any) side effects on plant growth.

The lack of any detectable decrease in grain weight, size, or starch content suggests that the lack of B-granules may not adversely affect yield. This contrasts with many other mutations known to disrupt cereal grain starch metabolism that, as well as changing starch granule morphology and/or composition, also result in reduced starch content and grain weight. In this respect, the *Bgc-1* mutation may be similar to mutations of bread wheat affecting granule-bound starch synthase I (GBSSI), that cause elimination of the amylose component of starch but which have a minimal effect on grain weight and yield (Graybosch *et al.*, 2003).

Near-normal yield together with novel grain and starch physicochemical properties indicate that B-less bread wheat may be commercially useful. For example, uniform and larger than normal starch granules may lead to improvements in the yield of purified starch and gluten (Stoddard and Sarker, 2000). Reduced grain hardness could lead to reduced milling energy (Greffeuille *et al.*, 2006). Increased swelling power could lead to improved eating quality of Japanese white salted noodles (Konik *et al.*, 1993). B-granules are also predicted to be detrimental for malting and distilling (Bathgate *et al.*, 1974; Tillett and Bryce, 1993), suggesting that B-less bread wheat may be preferred for alcohol production. We intend to test further the functional properties of B-less wheat and its utility in different manufacturing processes once sufficient materials are available.

Herein, we have described the deletion of a locus, *Bgc-1* in bread wheat (*T. aestivum*), whose existence and location in the genome were inferred from work on wild wheat (*Aegilops* spp.). Similar approaches include the direct introgression of genes/traits from wild species to bread wheat, as in Nemeth *et al.* (2015), and the identification of genes common to both types of wheat by positional cloning of the wild wheat gene, as in Yan *et al.* (2003). The Paragon deletion mutant population was also used previously to identify the *Ph1* locus in bread wheat (Al-Kaff *et al.*, 2008). However, we are not aware of any other work that has used precisely the same approach as that described here to identify and modify a trait in bread wheat.

## Supplementary data

Supplementary data are available at *JXB* online.

Fig. S1. Genotyping using marker KT71, by separation of PCR products by capillary electrophoresis.

Fig. S2. Exome sequencing data for Chr 1B, line A1 with data for four genes close to the deletion borders.

Fig. S3. Exome sequencing data for Chr 4A, line A1 with data for four genes close to the deletion borders.

Fig. S4. Exome sequencing data for Chr 5D, line A1 with data for four genes close to the deletion borders.

Fig. S5. Exome sequencing data for Chr 6A, line A1 with data for four genes close to the deletion borders.

Fig. S6. Exome sequencing data for Chr 1A, line D4 with data for four genes close to the deletion borders.

Fig. S7. Exome sequencing data for Chr 1B, line D4 with data for four genes close to the deletion borders.

Fig. S8. Exome sequencing data for Chr 4B, line D4 with data for four genes close to the deletion borders.

Fig. S9. Exome sequencing data for Chr 4D, line D4 with data for four genes close to the deletion borders.

Fig. S10. Exome sequencing data for Chr 6D, line D4 with data for four genes close to the deletion borders.

Fig. S11. Exome sequencing data for Chr 7A, line D4 with data for four genes close to the deletion borders.

Table S1. Oligonucleotide primers for PCR.

Table S2. Physical map positions of markers used for genotyping deletions.

## Supplementary Material

supplementary_figures

supplementary_tables
