# Supplementary material for: Transfer of a starch phenotype from wild wheat to bread wheat by deletion of a locus controlling B-type starch granule content
Source: J Exp Bot. 2017 Nov 1;68(20):5497–509. doi: 10.1093/jxb/erx349 (PMC5853964; doi:10.1093/jxb/erx349)
Supplement: supplementary_figures [file erx349_suppl_supplementary_figures.pdf]

Supplementary Figures.

Figure S1. Genotyping using marker KT7: by separation of PCR products by capillary electrophoresis.

Figures S2. Exome capture data for Chr 1B, line A1 with data for 4 genes close to the deletion borders.

Figures S3. Exome capture data for Chr 4A, line A1 with data for 4 genes close to the deletion borders.

Figures S4. Exome capture data for Chr 5D, line A1 with data for 4 genes close to the deletion borders.

Figures S5. Exome capture data for Chr 6A, line A1 with data for 4 genes close to the deletion borders.

Figures S6. Exome capture data for Chr 1A, line D4 with data for 4 genes close to the deletion borders.

Figures S7. Exome capture data for Chr 1B, line D4 with data for 4 genes close to the deletion borders.

Figures S8. Exome capture data for Chr 4B, line D4 with data for 4 genes close to the deletion borders.

Figures S9. Exome capture data for Chr 4D, line D4 with data for 4 genes close to the deletion borders.

Figures S10. Exome capture data for Chr 6D, line D4 with data for 4 genes close to the deletion borders.

Figures S11. Exome capture data for Chr 7A, line D4 with data for 4 genes close to the deletion borders.

Figure S1. Genotyping Marker KT71.

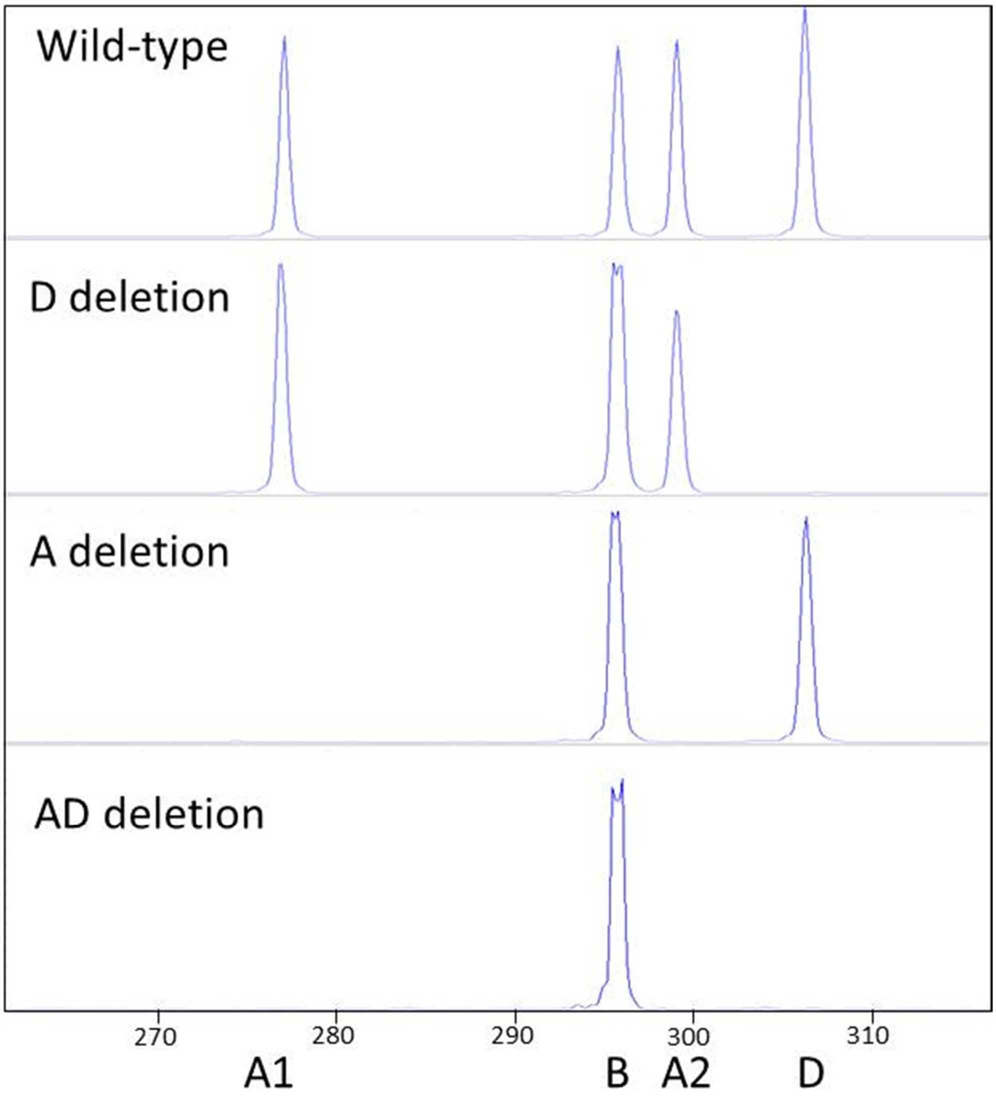

Figure S2.

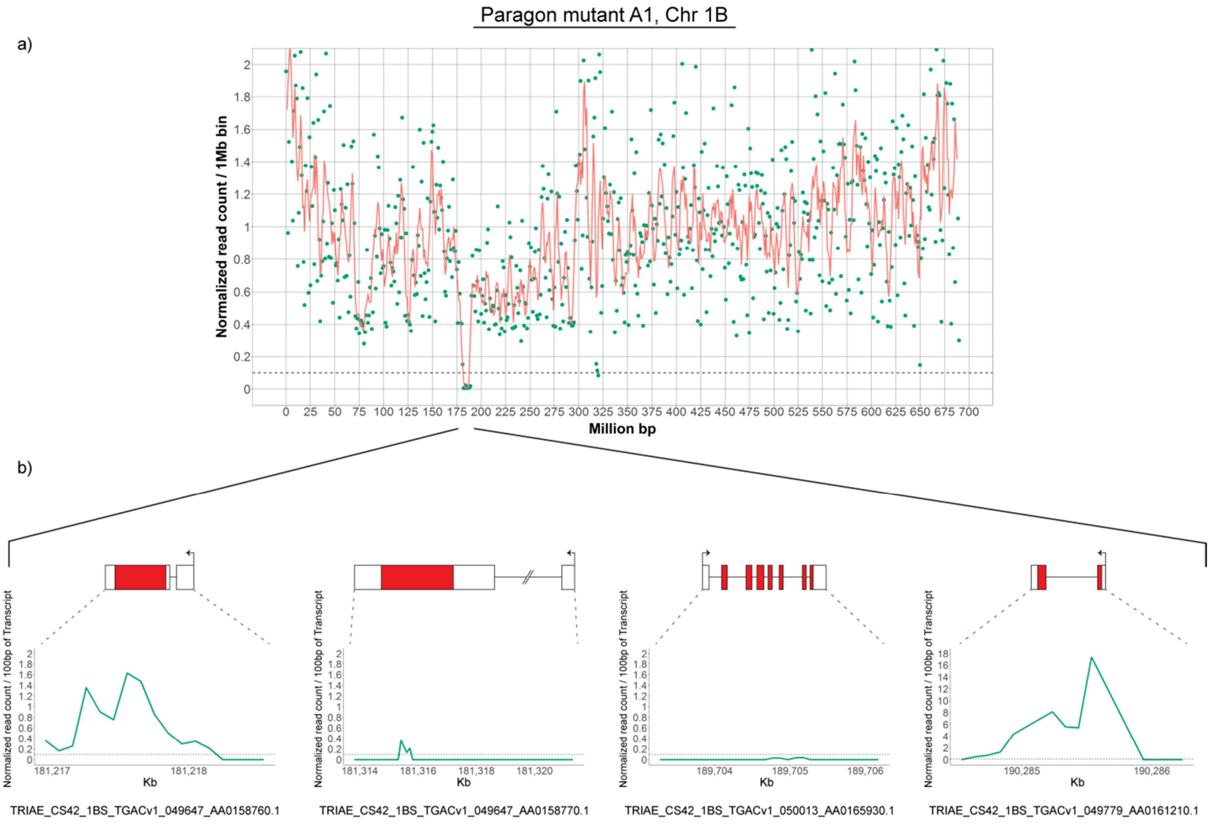

Paragon mutant A1, Chr 1B
a)
Normalized read count / 1Mb bin
Million bp
b)
Normalized read count / 100bp of Transcript
Kb
181,217
181,218
181,314
181,316
181,318
181,320
189,704
189,705
189,706
190,285
190,286
TRIAE_CS42_1BS_TGACv1_049647_AA0158760.1
TRIAE_CS42_1BS_TGACv1_049647_AA0158770.1
TRIAE_CS42_1BS_TGACv1_050013_AA0165930.1
TRIAE_CS42_1BS_TGACv1_049779_AA0161210.1

Figure S3

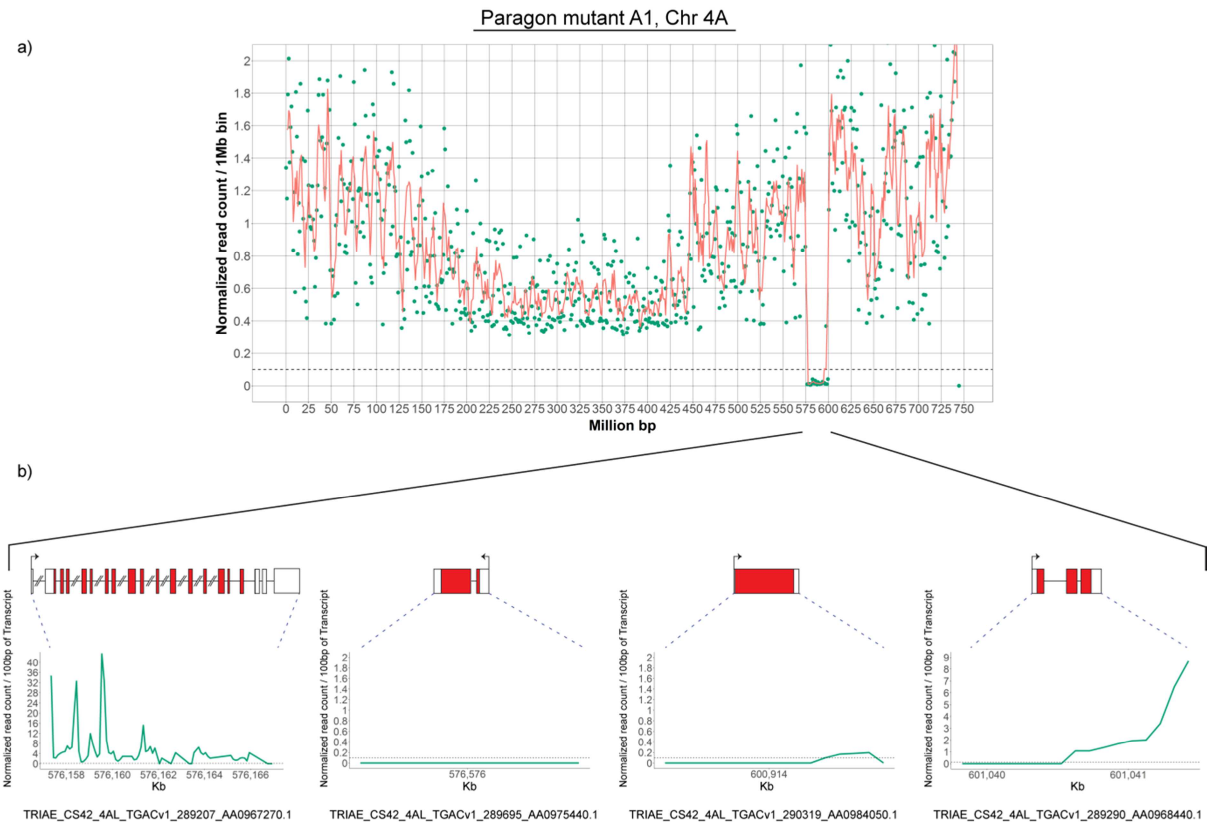

a)
Paragon mutant A1, Chr 4A
Normalized read count / 1Mb bin
Million bp
b)
Normalized read count / 100bp of Transcript
Kb
TRIAE_CS42_4AL_TGACv1_289207_AA0967270.1
TRIAE_CS42_4AL_TGACv1_289695_AA0975440.1
TRIAE_CS42_4AL_TGACv1_290319_AA0984050.1
TRIAE_CS42_4AL_TGACv1_289290_AA0968440.1

Figure S4

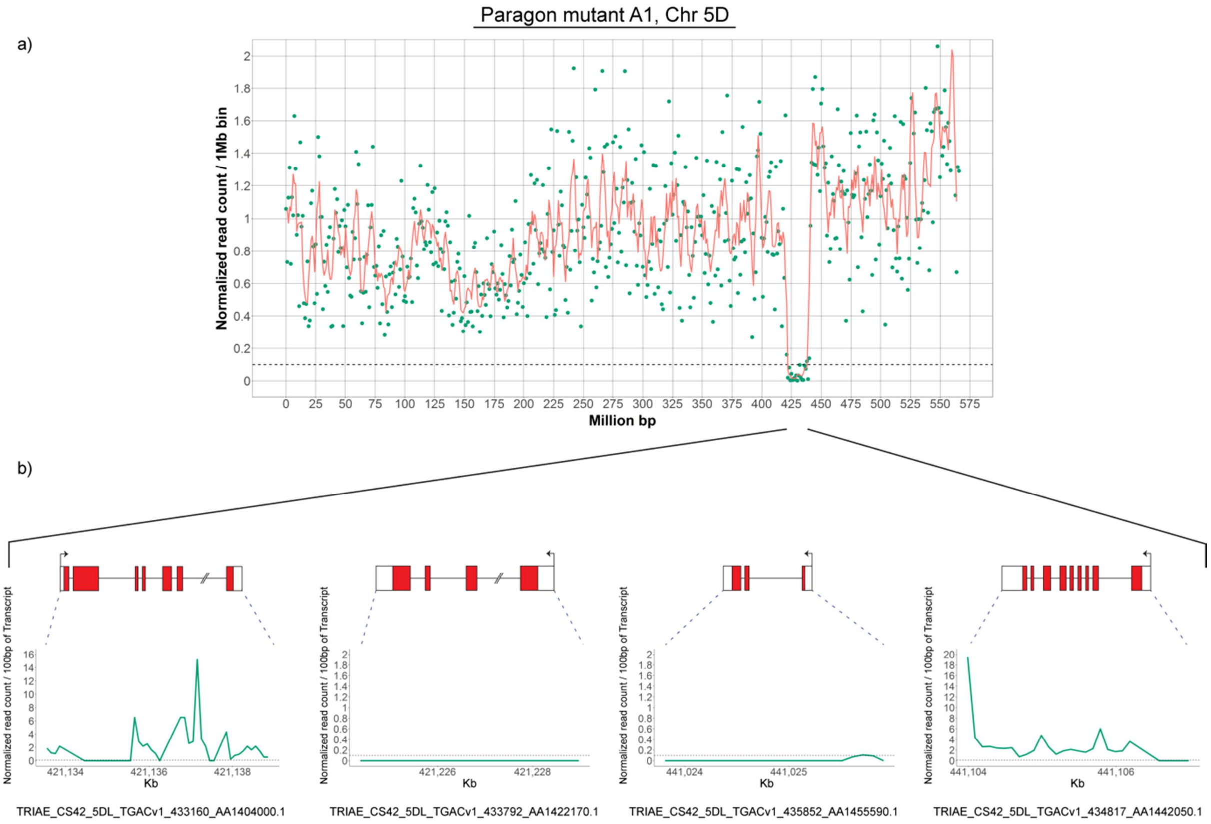
Paragon mutant A1, Chr 5D
a)
Normalized read count / 1Mb bin
Million bp
b)
Normalized read count / 100bp of Transcript
Kb
TRIAE_CS42_5DL_TGACv1_433160_AA1404000.1
TRIAE_CS42_5DL_TGACv1_433792_AA1422170.1
TRIAE_CS42_5DL_TGACv1_435852_AA1455590.1
TRIAE_CS42_5DL_TGACv1_434817_AA1442050.1

Figure S5

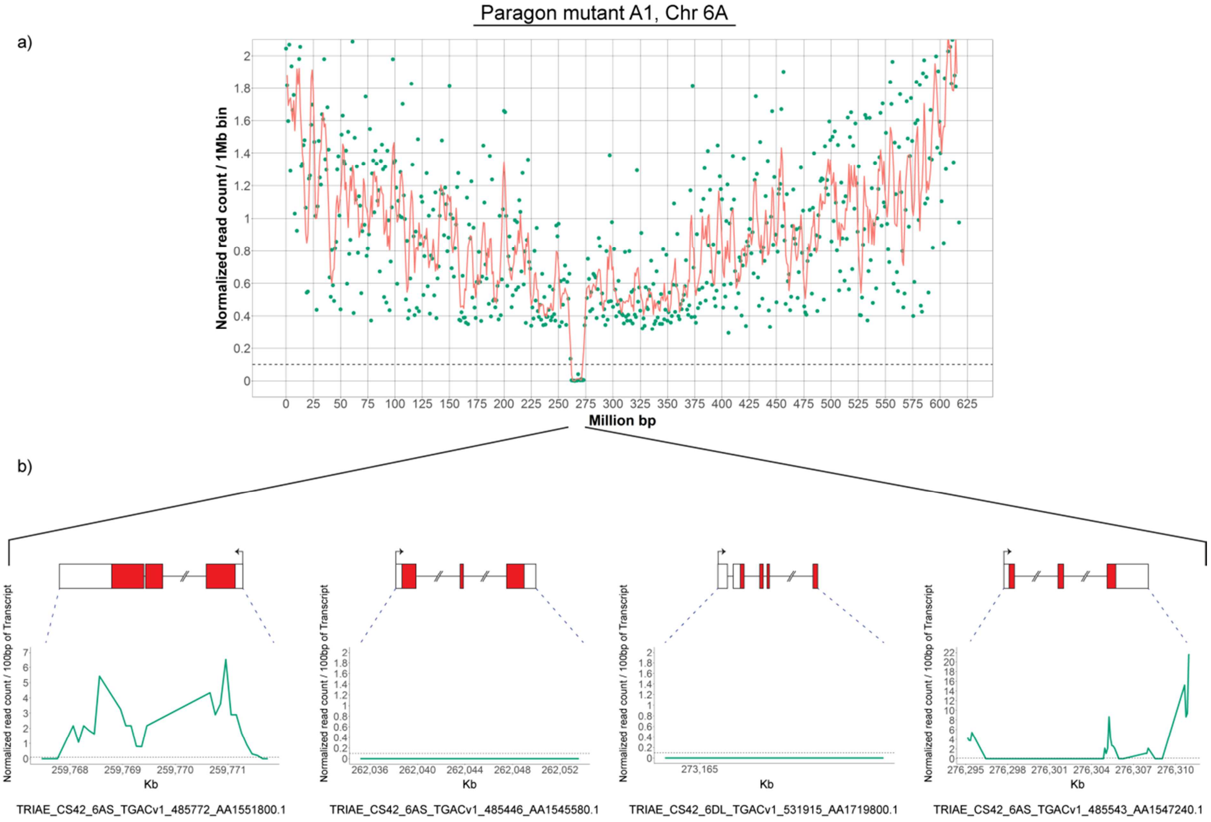

a)
Paragon mutant A1, Chr 6A
Normalized read count / 1Mb bin
Million bp
b)
Normalized read count / 100bp of Transcript
Kb
TRIAE_CS42_6AS_TGACv1_485772_AA1551800.1
TRIAE_CS42_6AS_TGACv1_485446_AA1545580.1
TRIAE_CS42_6DL_TGACv1_531915_AA1719800.1
TRIAE_CS42_6AS_TGACv1_485543_AA1547240.1

Figure S6.

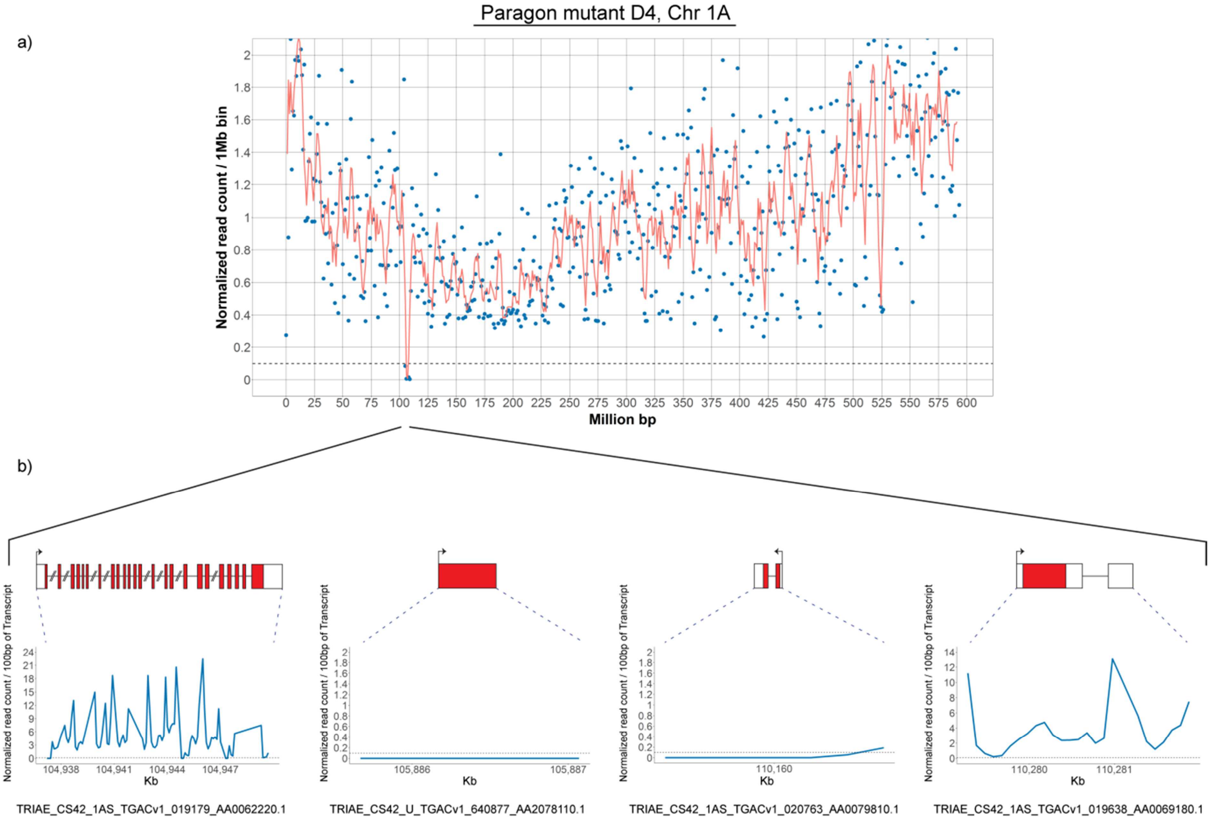

a)
Paragon mutant D4, Chr 1A
Normalized read count / 1Mb bin
Million bp
b)
Normalized read count / 100bp of Transcript
Kb
104,938
104,941
104,944
104,947
105,886
105,887
110,160
110,280
110,281
TRIAE_CS42_1AS_TGACv1_019179_AA0062220.1
TRIAE_CS42_U_TGACv1_640877_AA2078110.1
TRIAE_CS42_1AS_TGACv1_020763_AA0079810.1
TRIAE_CS42_1AS_TGACv1_019638_AA0069180.1

Figure S7.

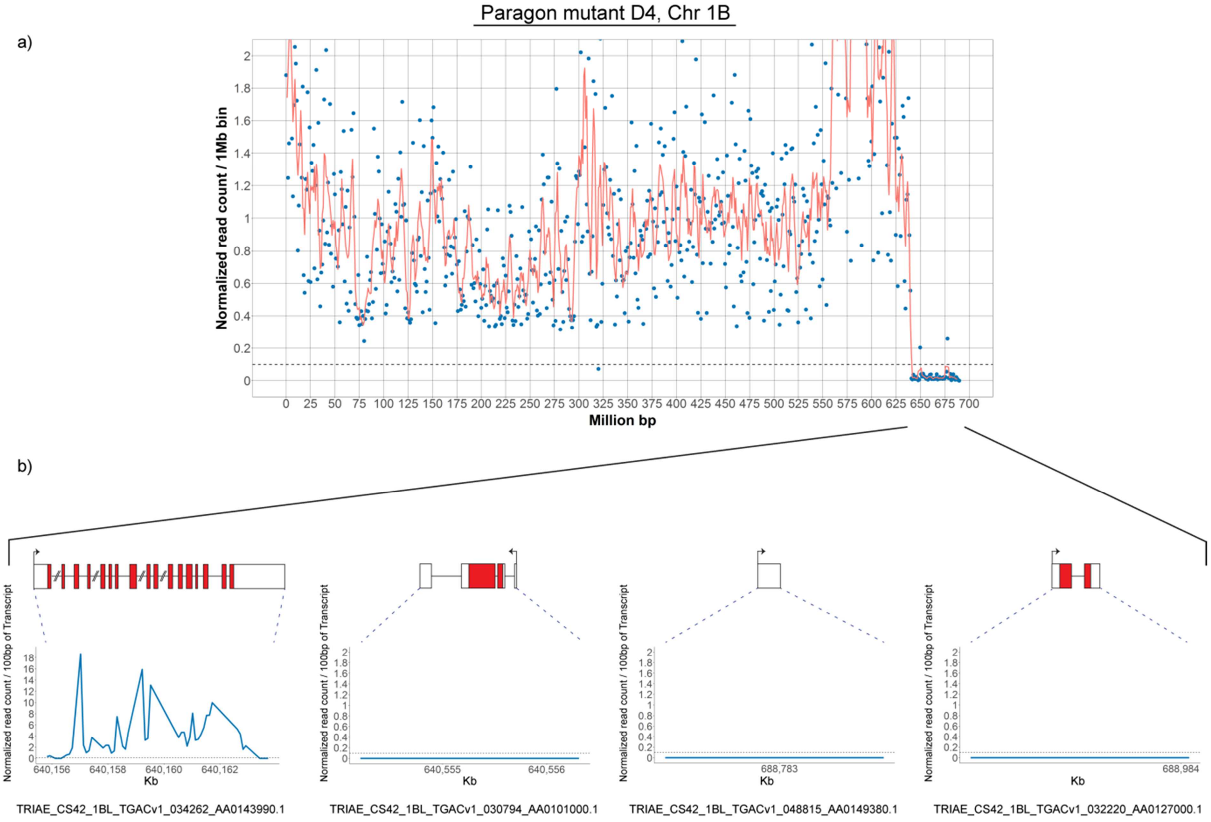
a)
Paragon mutant D4, Chr 1B
Normalized read count / 1Mb bin
Million bp
b)
Normalized read count / 100bp of Transcript
Kb
640,156
640,158
640,160
640,162
640,555
640,556
688,783
688,984
TRIAE_CS42_1BL_TGACv1_034262_AA0143990.1
TRIAE_CS42_1BL_TGACv1_030794_AA0101000.1
TRIAE_CS42_1BL_TGACv1_048815_AA0149380.1
TRIAE_CS42_1BL_TGACv1_032220_AA0127000.1

Figure S8.

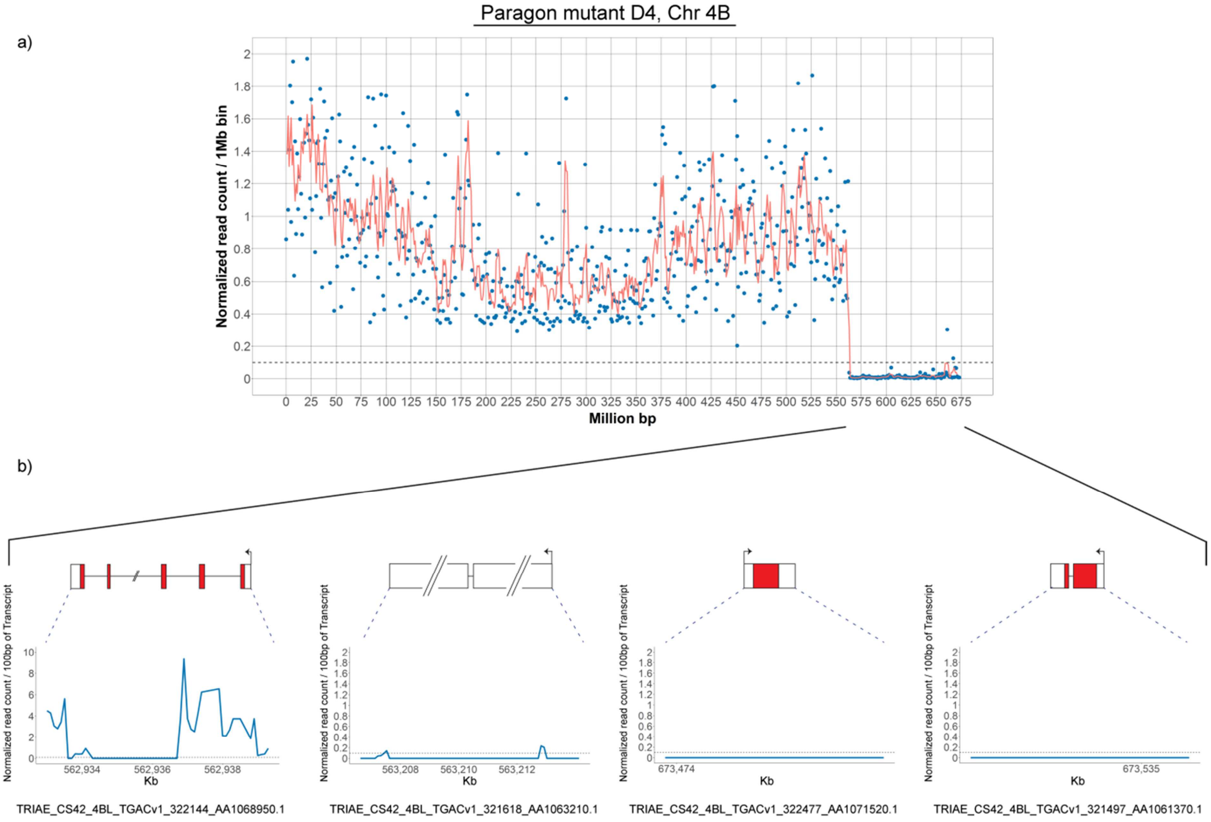
a)
Paragon mutant D4, Chr 4B
Normalized read count / 1Mb bin
Million bp
b)
Normalized read count / 100bp of Transcript
Kb
562,934
562,936
562,938
563,208
563,210
563,212
673,474
673,535
TRIAE_CS42_4BL_TGACv1_322144_AA1068950.1
TRIAE_CS42_4BL_TGACv1_321618_AA1063210.1
TRIAE_CS42_4BL_TGACv1_322477_AA1071520.1
TRIAE_CS42_4BL_TGACv1_321497_AA1061370.1

Figure S9.

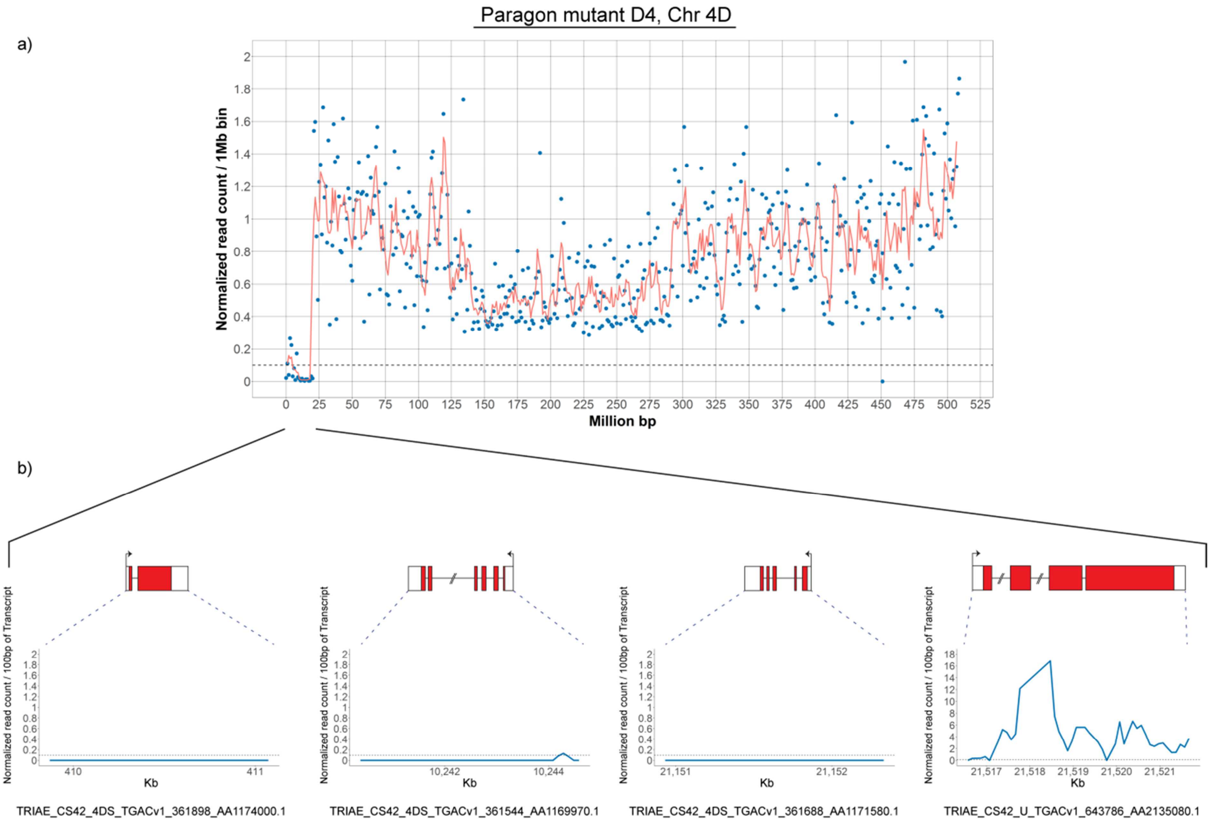
Paragon mutant D4, Chr 4D
a)
Normalized read count / 1Mb bin
Million bp
b)
Normalized read count / 100bp of Transcript
Kb
TRIAE_CS42_4DS_TGACv1_361898_AA1174000.1
TRIAE_CS42_4DS_TGACv1_361544_AA1169970.1
TRIAE_CS42_4DS_TGACv1_361688_AA1171580.1
TRIAE_CS42_U_TGACv1_643786_AA2135080.1

Figure S10.

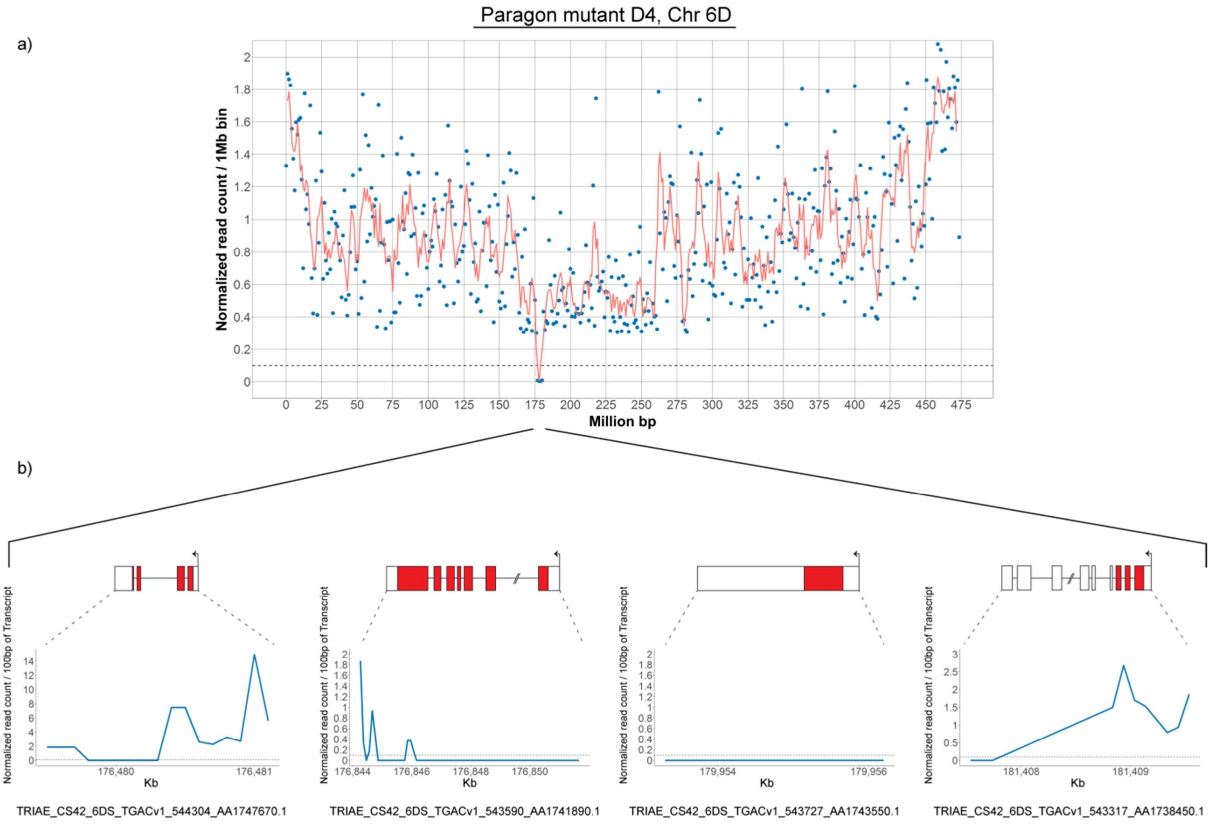

Paragon mutant D4, Chr 6D
a)
Normalized read count / 1Mb bin
Million bp
b)
Normalized read count / 100bp of Transcript
Kb
TRIAE_CS42_6DS_TGACv1_544304_AA1747670.1
TRIAE_CS42_6DS_TGACv1_543590_AA1741890.1
TRIAE_CS42_6DS_TGACv1_543727_AA1743550.1
TRIAE_CS42_6DS_TGACv1_543317_AA1738450.1

Figure S11.

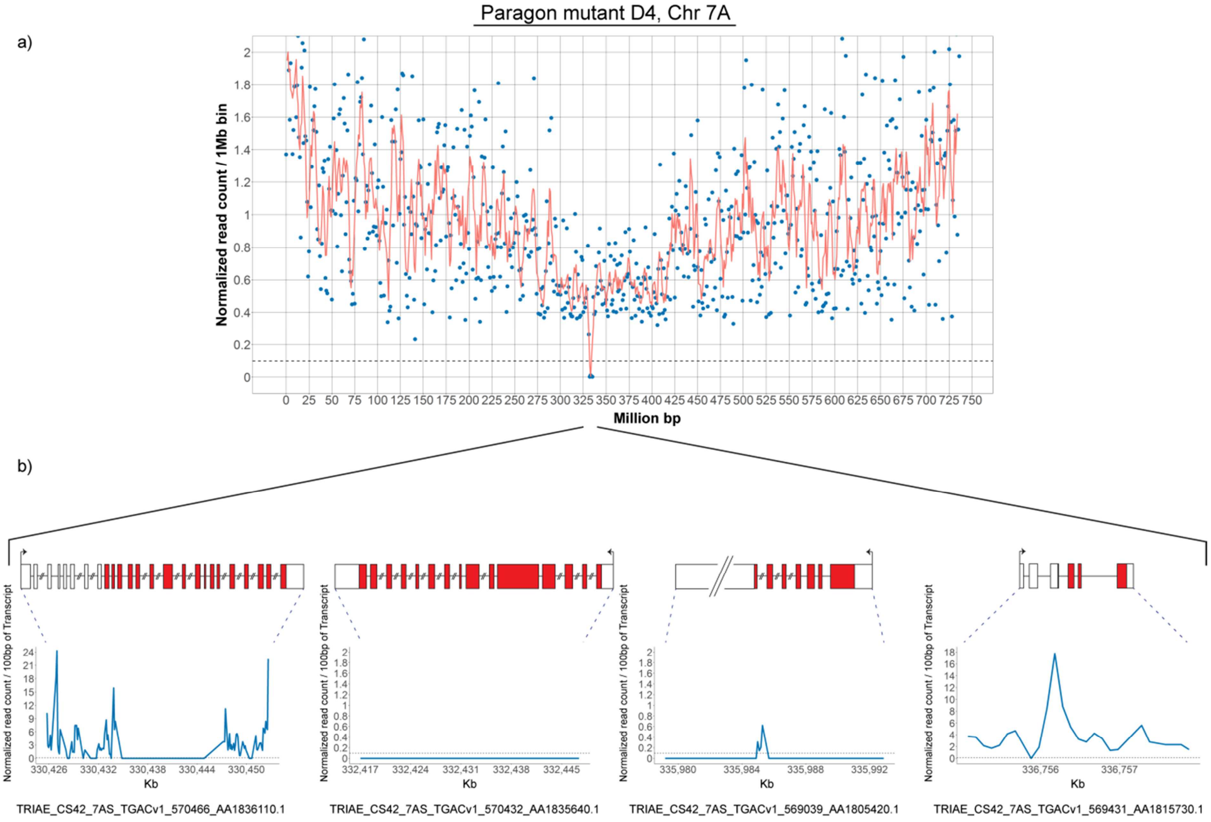
Paragon mutant D4, Chr 7A
a)
Normalized read count / 1Mb bin
Million bp
b)
Normalized read count / 100bp of Transcript
Kb
TRIAE_CS42_7AS_TGACv1_570466_AA1836110.1
TRIAE_CS42_7AS_TGACv1_570432_AA1835640.1
TRIAE_CS42_7AS_TGACv1_569039_AA1805420.1
TRIAE_CS42_7AS_TGACv1_569431_AA1815730.1
