# Supplementary material for: Transfer of a starch phenotype from wild wheat to bread wheat by deletion of a locus controlling B-type starch granule content
Source: J Exp Bot. 2017 Nov 1;68(20):5497–509. doi: 10.1093/jxb/erx349 (PMC5853964; doi:10.1093/jxb/erx349)
Supplement: supplementary_tables [file erx349_suppl_supplementary_tables.pdf]

Table S1. Oligonucleotide primers for PCR

Aegilops marker	Chromosome arm	Wheat gene orthologue	Primer Forward 5'-3'	Primer Reverse 5'-3'	Amplicon size (bp)	Anealing temp. (°C)	Extension time (s)	DMSO
TC37B	4AL	TRIAE_CS42_4AL_TGACv1_289950_AA0979330	AACTACAGATTCATGACAGG	CTACTGCTACCTCCTCTCTT	85	60	30s	No
	4BS	TRIAE_CS42_4BS_TGACv1_328956_AA1096100	-	-	-	-	-	-
	4DS	TRIAE_CS42_4DS_TGACv1_361737_AA1172310	ACGGATTAGTCACAACAAGC	GTTGGGAAGACAGATAATGC	80	60	30s	No
KT71	4AL	TRIAE_CS42_4AL_TGACv1_288750_AA0957220	GGARTTTGATTTCCCGCCAT	[FAM]GCAGCCCAGAAGAAAATGACA	279	59		No
	4AL	TRIAE_CS42_4AL_TGACv1_290111_AA0981820			301			
	4BS	TRIAE_CS42_4BS_TGACv1_328355_AA1086780			298			
	4DS	TRIAE_CS42_4DS_TGACv1_361106_AA1161080			310			
TC30B	4AL	TRIAE_CS42_4AL_TGACv1_290077_AA0981360	ATCTGGTACCTGATTTCATAGTGA	CCACAACTGTACCATTATCTACTGC	265	60	30s	Yes
	4BS	TRIAE_CS42_4BS_TGACv1_328641_AA1	CAAGGACGCAATCTCACCA	GCAACGAGGAGATGAGCC	506	60	30s	Yes

		091570						
	4DS	TRIAE_CS42_4DS_TGACv1_361093_AA1160690	CAAGTTCTCTACGGTTTGGAGT	TTGATCAAGAGAATGGGGAT	311	60	30s	Yes
TC34	4AL	TRIAE_CS42_4AL_TGACv1_289509_AA0972250	ATGACACCTTTATTTCAGCCAG	AGCTCGGTCTGCATTTGA	639	62	30s	No
	4BS	TRIAE_CS42_4BS_TGACv1_328517_AA1089200	CGCTCACCATCACCCAAG	GAGCTGAAGCGAACGAAC	283	62	30s	No
	4DS	TRIAE_CS42_4DS_TGACv1_362688_AA1181550	TGCCATCATCGGTAGTCATT	TGGTGTGCTGTTGATCCTT	333	62	30s	No
4G	4AL	TRIAE_CS42_4AL_TGACv1_289219_AA0967530	GATGAGCCGCCTCCCCAT	CCTTTGCTGATGCAGTTCG	304	60	30s	No
	4BS	TRIAE_CS42_4BS_TGACv1_330068_AA1105770	-	-	-	-	-	-
	4DS	TRIAE_CS42_4DS_TGACv1_362384_AA1179360	TGGAACACTGCCATCGTG	GGTGGAGCGAGATATGAGATC	311	60	30s	No
4N5.5	4DS	TRIAE_CS42_4DS_TGACv1_363571_AA1183970	GGCTTTGATACTGGAACGAAT	CAGTGTAAGGCTCTGTTGCG	-	-	-	-

Primers were designed to wheat TGACv1 cv. Chinese Spring gene models (Clavijo et al., 2017). Marker KT71 was designed to amplify all homoeologues whilst the other markers were designed to be homoeologue-specific. For 4N5.5, only a D-genome-specific marker was designed. FastStart Taq DNA Polymerase and buffer (Roche supplied by Sigma-Aldrich) were used in PCR reactions according to the manufacturer's instructions. Primer sequences and PCR conditions are shown above. [FAM] = 5' FAM (6-fluorescein amidite) labelling of primer.

Table S2. Physical map positions of markers used for genotyping deletions

	Chromosome arm	Wheat gene orthologue/deletion	Position on 4AL (bp)		Position on 4DS (bp)	
			**start**	**end**	**start**	**end**
**Deletion (exome capture data)**						
	4AL		576,167,498	601,039,825		
	4DS				0	21,516,171
***Aegilops* markers**						
TC37B	4AL	TRIAE_CS42_4AL_TGACv1_289950_AA0979330	591,845,338	591,852,359		
	4DS	TRIAE_CS42_4DS_TGACv1_361737_AA1172310			10,966,857	10,975,050
KT71	4AL	TRIAE_CS42_4AL_TGACv1_288750_AA0957220	590,187,733	590,179,759		
	4AL	TRIAE_CS42_4AL_TGACv1_290111_AA0981820	590,119,776	590,111,029		
	4DS	TRIAE_CS42_4DS_TGACv1_361106_AA1161080			12,457,943	12,465,627
TC30B	4AL	TRIAE_CS42_4AL_TGACv1_290077_AA0981360	589,607,862	589,602,306		
	4DS	TRIAE_CS42_4DS_TGACv1_361093_AA1160690			12,704,776	12,699,536
TC34	4AL	TRIAE_CS42_4AL_TGACv1_289509_AA0972250	589,010,812	589,016,280		
	4DS	TRIAE_CS42_4DS_TGACv1_362688_AA1181550			13,248,110	13,242,447
4G	4AL	TRIAE_CS42_4AL_TGACv1_289219_AA0967530	584,700,513	584,693,518		
	4DS	TRIAE_CS42_4DS_TGACv1_362384_AA1179360			15,877,787	15,870,454
4N5.5	4DS	TRIAE_CS42_4DS_TGACv1_363571_AA1183970			16,611,579	16,608,549

The positions of the deletions in cv. Paragon deletion lines A1 and D4, as determined by exome capture and of the genes used as PCR markers (IWGSC RefSeq v1.0, cv. Chinese spring) are shown.
